# Varicella Pneumonia in an Immunocompetent Middle-aged Adult Male: A Case Report

**DOI:** 10.31729/jnma.7427

**Published:** 2022-07-31

**Authors:** Nischal Shrestha, Karuna Bhatta, Anjan Rai, Sanzida Khatun

**Affiliations:** 1 Department of Internal Medicine, Nobel Medical College Teaching Hospital (P) Ltd., Biratnagar, Morang, Nepal; 2 Department of Pulmonary, Critical Care and Sleep Medicine, Nobel Medical College Teaching Hospital (P) Ltd., Biratnagar, Morang, Nepal; 3 Department of Dermatology, Nobel Medical College Teaching Hospital (P) Ltd., Biratnagar, Morang, Nepal; 4 Department of Anatomy, Nobel Medical College Teaching Hospital (P) Ltd., Biratnagar, Morang, Nepal

**Keywords:** *chickenpox*, *pneumonia*, *skin eruptions*

## Abstract

Varicella pneumonia is uncommon among adults and can present as potentially life-threatening complications of varicella. Here we report a case of a 43-year-old man with no known history of chronic disease and no allergic history who presented to our hospital emergency department with widespread skin eruptions over the entire body and hemoptysis. Varicella pneumonia was diagnosed based on the patient being in contact with his 6-year-old son who had contracted chickenpox 10 days back, typical cutaneous lesions, pulmonary symptoms and radiographic findings. The patient was treated with oral acyclovir and was admitted to the intensive care unit for monitoring. The patient recovered completely after 10 days of treatment.

## INTRODUCTION

Varicella pneumonia is a serious complication of chickenpox which is caused by varicella zoster virus (VZV), part of the Herpesviridae family or its reactivation. It is estimated to occur in 1 out of 400 chickenpox infections (<1% of cases) with the incidence in adults varying between 0.32-1.36 cases per 100,000 persons per year with mortality ranging from 10 to 30%.^1^ VZV spreads to the lung through hematogenous route.^2^ Though unusual but as a consequence of high mortality, this type of case requires more caution. Here, we present a case of a varicella pneumonia in an adult.

## CASE REPORT

A 43-year-old male, a construction worker, smoker for 25 years (1/2 pack of cigarettes per day, approximately 12.5 pack-years) presented in the emergency room (ER) of a tertiary care centre on the 4^th^ day following the first dose of COVID-19 vaccine with chief complaints of vesicular skin rash all over the body approximately 1 hour after vaccination, coughing out blood mixed sputum and shortness of breath on exertion. The patient gave a history of the sudden appearance of rashes all over the body as well as oral mucosa except palm and soles. The patient had a mild fever since the morning before the rashes and the fever continued to rise with a high grade documented as 38.33°C a few hours after the rashes. He took paracetamol but the fever reappeared the next day and it subsided only on the 3^rd^ day. He had sudden cough approximately 8 hours after the appearance of rashes with scanty blood mixed sputum in every episode of coughing. His 6-year-old son had been diagnosed with chickenpox 10 days back. The patient had no history of chickenpox previously and had not been immunised with the varicella vaccine. He gave no past history of hypertension (HTN), type 2 diabetes mellitus (DM), asthma, tuberculosis and thyroid disease. Reverse transcriptase-polymerase chain reaction (RT-PCR) for severe acute respiratory syndrome coronavirus 2 (SARS-CoV-2) from his nasopharyngeal swabs was negative on the 4^th^ day of rashes. He gave no history of regular intake of any medication, allergy and gastroesophageal reflux disease (GERD).

**Figure 1:**
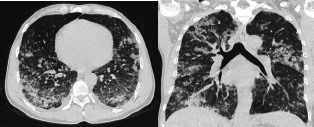
HRCT chest shows multiple areas of ground-glass opacities with a crazy-paving pattern, centrilobular nodules in branching pattern giving the appearance of tree in bud appearance.

On presentation to the ER, he was hypotensive (100/60 mm of Hg), tachycardia (106 beats/min), tachypneic (24 breaths/min), and afebrile (36.67°C) and his oxygen saturation was 88% in room air. He had crackles in both lung bases. He had a polymorphic rash with vesicles, pustules and crusty lesions. High-resolution computed tomography (HRCT) showed multiple areas of ground-glass opacities with a crazy-paving pattern, and centrilobular nodules in a branching pattern giving the appearance of a tree in bud appearance (Figure 1).

**Figure 2:**
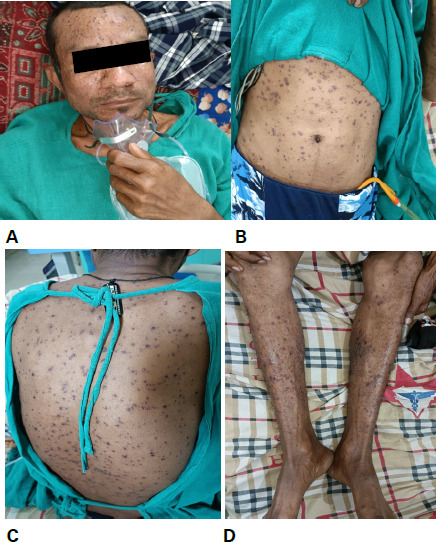
Multiple skin rashes (papules, vesicles and few crusts) over the A) face; B) trunk; C) back; D) legs.

The diagnosis of varicella pneumonia was made based on the presence of the pulmonary symptoms, contact history and typical skin rashes (Figure 2).

The patient was treated with oral acyclovir 800 mg for 7 days and admitted to the intensive care unit for further monitoring and later to the pulmonary ward after 3 days. The patient then recovered with complete resolution of the lung lesions, and he was discharged.

## DISCUSSION

Chicken pox is more common in children than in adults. If chickenpox develops in adults, pulmonary complications will develop in 5% to 15% of cases.^3^ Typically, pulmonary symptoms like cough, dyspnea, chest pain, difficulty in breathing and hemoptysis develop 1 to 6 days after the onset of skin rash.^4^ But in this case, hemoptysis occurred just a few hours after skin eruption. Few studies have reported pulmonary symptoms before the appearance of rashes.^5-7^ Risk factors for the development of pneumonia in chickenpox infection include pregnancy, age, smoking, chronic obstructive pulmonary disease, and immunosuppression.^8^ The patient in this study is a chronic smoker. One study reported that adult chickenpox cases developed pneumonia in 36.8% of smokers and in approximately 3% of non-smokers.^5^

Chickenpox is a clinical diagnosis but confirmatory diagnosis can be done by PCR from vesicle fluid.^9^ But PCR test for such a case was not available in our hospital. Usually, the radiological findings are nodular opacities involving all lung fields.^10^ Similar findings was observed in the HRCT chest in our case. The first case of treatment of varicella pneumonia with acyclovir was reported in 1980.^11^ At present, it has become standard therapy for patients with varicella infection. Early therapy with oral acyclovir decreases the time for healing skin rashes and another symptomatic improvement. However, initiation of therapy after >24 hours in an uncomplicated case of adult varicella is of no use.^12^ But our case presented on the 4^th^ day of rash and we started oral acyclovir the patient's symptoms improved and the patient was discharged on the 10^th^ day of admission.

In conclusion, chickenpox is rare in adults and varicella pneumonia, though unusual, is a serious complication. As signs and symptoms of pneumonia can occur before the onset of skin rashes, it is difficult to diagnose it as varicella pneumonia first-hand. As the patient gave a history of the appearance of skin rashes an hour after the COVID-19 vaccination, the vaccine could have hastened the disease process or it could be just a coincidence as this type of clinical picture has not been reported in the literature before.
